# Integrating Sexual and Reproductive Health Equity Into Public Health Goals and Metrics: Comparative Analysis of Healthy People 2030’s Approach and a Person-Centered Approach to Contraceptive Access Using Population-Based Data

**DOI:** 10.2196/58009

**Published:** 2024-08-20

**Authors:** Anu Manchikanti Gomez, Reiley Diane Reed, Ariana H Bennett, Megan Kavanaugh

**Affiliations:** 1 School of Social Welfare University of California, Berkeley Berkeley, CA United States; 2 Guttmacher Institute New York, NY United States

**Keywords:** contraception, public health objectives, public health metrics, person-centeredness, sexual and reproductive health equity

## Abstract

**Background:**

The Healthy People initiative is a national effort to lay out public health goals in the United States every decade. In its latest iteration, Healthy People 2030, key goals related to contraception focus on increasing the use of effective birth control (contraceptive methods classified as most or moderately effective for pregnancy prevention) among women at risk of unintended pregnancy. This narrow focus is misaligned with sexual and reproductive health equity, which recognizes that individuals’ self-defined contraceptive needs are critical for monitoring contraceptive access and designing policy and programmatic strategies to increase access.

**Objective:**

We aimed to compare 2 population-level metrics of contraceptive access: a conventional metric, use of contraceptive methods considered most or moderately effective for pregnancy prevention among those considered at risk of unintended pregnancy (approximating the Healthy People 2030 approach), and a person-centered metric, use of preferred contraceptive method among current and prospective contraceptive users.

**Methods:**

We used nationally representative data collected in 2022 to construct the 2 metrics of contraceptive access; the overall sample included individuals assigned female at birth not using female sterilization or otherwise infecund and who were not pregnant or trying to become pregnant (unweighted N=2760; population estimate: 43.9 million). We conducted a comparative analysis to examine the convergence and divergence of the metrics by examining whether individuals met the inclusion criteria for the denominators of both metrics, neither metric, only the conventional metric, or only the person-centered metric.

**Results:**

Comparing the 2 approaches to measuring contraceptive access, we found that 79% of respondents were either included in or excluded from both metrics (reflecting that the metrics converged when individuals were treated the same by both). The remaining 21% represented divergence in the metrics, with an estimated 5.7 million individuals who did not want to use contraception included only in the conventional metric denominator and an estimated 3.5 million individuals who were using or wanted to use contraception but had never had penile-vaginal sex included only in the person-centered metric denominator. Among those included only in the conventional metric, 100% were content nonusers—individuals who were not using contraception, nor did they want to. Among those included only in the person-centered metric, 68% were currently using contraception. Despite their current or desired contraceptive use, these individuals were excluded from the conventional metric because they had never had penile-vaginal sex.

**Conclusions:**

Our analysis highlights that a frequently used metric of contraceptive access misses the needs of millions of people by simultaneously including content nonusers and excluding those who are using or want to use contraception who have never had sex. Documenting and quantifying the gap between current approaches to assessing contraceptive access and more person-centered ones helps clearly identify where programmatic and policy efforts should focus going forward.

## Introduction

### Overview

Reproductive well-being requires the ability to prevent, continue, and end pregnancy in line with one’s desires, values, and preferences. For many people, use of contraception is crucial to reproductive well-being, as it is a key tool that people can use to help them accomplish their self-defined reproductive goals. Programs and policies in the United States that are designed to facilitate contraceptive access should have people’s reproductive well-being as a north star and be explicitly guided by principles of sexual and reproductive health (SRH) equity. The SRH equity framework lays out a vision for the conditions required for individuals to attain their maximal state of SRH and well-being [1,2]. This vision includes attention to structural inequities—based on historical and current systems of oppression—and the ways in which they differentially affect and marginalize certain communities. Similarly, efforts to track the extent to which programs and policies are making progress toward contraceptive access goals should also be guided by SRH equity.

Person-centered contraceptive access refers to the opportunity to have self-defined contraceptive needs fulfilled [3]. Therefore, person-centered contraceptive access is not just about contraceptive use or the availability of services but also encompasses the formation and realization of preferences, including use of preferred contraceptive methods as well as the accessibility, affordability, and appropriateness of care to obtain desired contraception. One key national effort that sets public health goals related to contraceptive access is the US Healthy People initiative, which, each decade, lays out broad objectives with related targets and activities across multiple health domains to advance health and well-being. In Healthy People and many other public health efforts that focus on contraceptive access, goals and associated metrics typically emphasize contraceptive use over nonuse, often prioritizing use of certain methods and neglecting people’s contraceptive desires [4,5]. Importantly, there are no national-level public health goals that currently place individuals’ contraceptive needs—as they define them—at the center of their objectives and related tracking efforts. Without capturing this important aspect of contraceptive use and access, efforts to conduct surveillance and design strategies to increase contraceptive access are severely constrained and do not take a person-centered approach. Furthermore, without person-centered objectives and data to track progress against, efforts to expand contraceptive access under current public health objectives and benchmarks may result in inefficiency and wasted resources and perpetuate contraceptive coercion and harm.

In this study, we considered 2 contraception-focused objectives and associated metrics of Healthy People 2030 (HP2030), the most recent national initiative guiding contraceptive access and other public health objectives at the national level in the United States. We highlighted key conceptual and empirical limitations embedded in the HP2030 contraception-related goals. Our analysis highlights shortcomings of conventional public health approaches and offers future directions to track and advance person-centered contraceptive access that align with SRH equity.

### A Primer on the Healthy People Initiative

The Healthy People initiative plays a significant role in shaping public health and policy in the United States by establishing a comprehensive list of health-related objectives in 10-year cycles. Surgeon General Julius Richmond launched the initiative in 1979, with the first set of objectives (Healthy People 1990) released in 1980 [6]. New objectives have been released in each decade since. While each update integrates lessons learned, Healthy People remains focused on its original mission of improving health outcomes in the United States. Released in August 2020, HP2030 reflects an evolution of the initiative to address health equity. HP2030 has a stated mission to “eliminate health disparities, achieve health equity, and attain health literacy to improve the health and well-being of all” [7]. All objectives are set by federal interdisciplinary expert working groups based on alignment with both federal and public health priorities, existing baseline data, and multi-sector stakeholder input via public comment [7].

### Limitations of HP2030’s Focus on Increasing Use of Effective Contraceptive Methods

#### Overview

HP2030 objectives related to contraception fall in the Family Planning topic area, which includes goals focused on improving pregnancy planning and preventing unintended pregnancy [8]. Many scholars have highlighted the problematic nature of the prevention of unintended pregnancy as a public health goal, emphasizing both the poor measurement and conceptualization of unintended pregnancy, as well as how continued focus on purportedly appropriate timing of childbearing perpetuates stratified reproduction [9-15]. In this paper, we focus more narrowly on 2 of HP2030’s core contraception-focused objectives, each centered on increasing use of effective birth control among those at risk of unintended pregnancy. The first objective focuses on increasing use among women aged 20 to 44 years [5], whereas the second focuses on adolescent female individuals aged 15 to 19 years [4].

As with all HP2030 objectives, these 2 contraception-related goals are connected to associated metrics to track progress. The numerator reflects use of effective contraceptive methods, defined as methods considered most or moderately effective at preventing pregnancy (permanent contraception, implant, intrauterine devices [IUDs], injectable contraception, oral contraceptive pills, the patch, the ring, or the diaphragm) [16]. Although the objectives refer to effective birth control, it is important to note that effectiveness is restricted to pregnancy prevention, neglecting that some methods are effective at achieving other user-desired outcomes (eg, preventing sexually transmitted infections or menstruation management). The denominator for both is the population of those at risk of unintended pregnancy, defined as women in each of the age ranges who have ever had sex with a man; are not pregnant, seeking pregnancy, or post partum; and are not sterile (for surgical noncontraceptive or nonsurgical reasons) [17]. Progress toward goals is tracked using the National Survey of Family Growth, a periodic, nationally representative survey used to monitor SRH, family and relationships, and health behaviors. The most recent 2017 to 2019 National Survey of Family Growth data indicate that 62.2% of women aged 20 to 44 years and 53.3% of adolescent female individuals aged 15 to 19 years at risk of unintended pregnancy are using effective contraception [4,5]. HP2030 has set a national goal of increasing use of effective contraception to 65.1% among those aged 20 to 44 years and 70.1% among those aged 15 to 19 years.

In the following sections, we highlight key conceptual and empirical limitations of these 2 HP2030 metrics. Furthermore, as the mission of HP2030 includes advancing health equity and eliminating health disparities, we sought to elucidate the alignment—or lack thereof—of these 2 objectives with the principles of SRH equity.

#### Issues With the Population of Focus

Both HP2030 metrics focus on individuals at risk of unintended pregnancy, which implicitly links contraceptive use to penile-vaginal sex and excludes use of contraception for reasons beyond pregnancy prevention. Extant research describes the many reasons individuals choose to use or not use contraception, including reasons completely delinked from sex and reproduction [18-21]. For example, in a 2023 national survey, 66% of those aged 15 to 29 years who had ever used hormonal contraception did so to manage menstruation-related symptoms, such as heavy bleeding and cramping [22]. Previous research elucidates that some people may not report actively seeking pregnancy but they may find it acceptable [23-26], that the notion of pregnancy planning is not always salient [27,28], and that individuals may not express explicit intentions because they lack the structural conditions to claim their underlying desires [11]. Furthermore, because of the focus on (presumed) risk of unintended pregnancy, the HP2030 metrics include in their denominators content nonusers—individuals not using contraception who do not want to use contraception. This conflicts with SRH equity by placing value on use of methods even when individuals prefer to use no method at all [1,29].

Notably, these 2 objectives explicitly name women and female adolescents as the populations of focus, specifically those who ever had sex (with a man) as a proxy for risk of pregnancy. Not all people who (want to) use contraception identify as women or female, some people who identify as men may not produce sperm, and having had sex (presumably penile-vaginal sex) at some point in one’s lifetime does not speak to current risk of pregnancy. As gender-expansive individuals already face increased stigma and discrimination in health care settings and must additionally navigate contraceptive care being provided under the umbrella of women’s health, this approach reiterates a cisnormative, heteronormative binary [30-33]. Notably, the data measurement details for each objective do not detail how gender identity is measured [17].

#### Lack of Person-Centeredness

Contraceptive use is a preference-sensitive decision, meaning that there is often no significant medical benefit to one choice versus another; therefore, there are multiple appropriate options for most individuals, including methods deemed as less effective for pregnancy prevention or other reasons [34]. However, the focus of these HP2030 objectives on increasing use of effective contraception neglects the preference-sensitive nature of contraceptive decision-making. By positioning use of most or moderately effective methods as successful, use of other methods or no method are thereby situated as a failure. This framing may inadvertently motivate family planning providers to encourage their patients to initiate and continue to use highly effective methods, especially long-acting reversible contraception, even in the absence of a patient’s desire to use these methods [35-40]. Such directive contraceptive counseling undermines reproductive autonomy and reinforces mistrust in health care, particularly among communities subjected to historical and ongoing medical coercion and abuse [41,42]. Furthermore, extensive research has documented that effectiveness for pregnancy prevention is only 1 factor among many that people consider when choosing the best contraceptive method for them [19,43-46].

Person-centered care, as defined by the Institute of Medicine, is “compassionate, respectful, and responsive to the needs, values, and expressed desires of each individual person” [47]. Setting population-level goals for use of specific groups of contraceptive methods without clear evidence that individuals desire them—as both HP2030 objectives do—ignores the needs, values, and desires that undergird the choice to use a specific method or to not use contraception. Furthermore, given that contraceptive use is a dynamic journey and US women use a median of 3 methods during their lifetimes [48], it is expected that individuals—including users of these effective methods—will start, switch, and discontinue methods throughout their lives [18,49,50]. For example, one may be using a method that requires a provider to remove it (eg, an implant) but desire to stop using it immediately. Through the lens of the HP2030 metrics, this individual’s use of a highly effective method is framed as a success and would contribute to meeting these targets even though they desire discontinuation. Designating use of these methods as a universally positive outcome without consideration of people’s own preferences is particularly concerning in light of research findings that Black and Latino individuals are more likely to prefer methods that they can start without seeing a health care provider [19] and less likely to receive person-centered contraceptive counseling [51].

### Use of Preferred Contraceptive Method as a Person-Centered Metric of Contraceptive Access

The ability to realize one’s contraceptive preferences represents one successful outcome in the cumulative process of navigating contraceptive access, from information seeking to desired contraceptive use [3]. Therefore, one person-centered metric of contraceptive access should capture the extent to which people are using the contraceptive method they prefer and be mindful that not using contraception may be preferred. As Burke and Potter [52] note, achieving contraceptive preferences is an indicator of reproductive autonomy and, therefore, can be considered to represent one aspect of success in achieving contraceptive access. In an analysis of population-based data, we found that 59.3% of current and prospective users (aged 15-44 years) in the United States were using a preferred contraceptive method [53].

Given the limitations of the HP2030 approach, use of person-centered contraceptive access metrics aligned with SRH equity is of paramount importance. Understanding the similarities and differences between the HP2030 approach and a person-centered approach highlights the implications of not centering contraceptive preferences—an important task given that population-level metrics of contraceptive access and quality worldwide generally do not align with SRH equity principles [29,52]. Therefore, in this analysis, we drew on nationally representative survey data to compare a conventional metric of contraceptive access approximating the HP2030 metrics (ie, use of effective contraception) to a person-centered metric (ie, use of preferred contraceptive method). With this analysis, we sought to compare SRH and demographic characteristics among the groups that are included in and excluded from the 2 metrics as well as elucidate the contraceptive use preferences of respondents included in the conventional measurement approach that implicitly frames use of effective contraceptive methods as successful. In so doing, we examined the assumptions built into the HP2030 objectives and considered their implications for public health policy and practice and for advancing SRH equity.

## Methods

### Overview

We used data collected through the Person-Centered Contraceptive Access Metrics Project, a multiyear, stakeholder-engaged effort to develop new population-level metrics of contraceptive access grounded in person-centeredness and reproductive justice [53,54]. Through a multidisciplinary working group that formulated the metrics, this project built upon the wisdom and expertise of stakeholders experienced in numerous sectors who produce, use, or would like to use contraceptive access metrics. The group developed the metric of use of preferred method of contraception to capture self-defined contraceptive need, described in the following sections [53].

### Data Source

We used nationally representative survey data collected between January 2022 and March 2022 via the AmeriSpeak panel by NORC at the University of Chicago [55]. AmeriSpeak is a probability-based standing survey panel that is representative of the US population. Eligible panelists were aged 15 to 44 years, assigned female sex at birth, not known to be sterile, and could complete the survey in English or Spanish. The median survey completion time was 25 minutes. Approximately 97% of screened eligible panelists completed the survey (unweighted N=3059). Further details about the survey methods in accordance with the Checklist for Reporting Results of Internet E-Surveys can be found in Multimedia Appendix 1 [56].

### Ethical Considerations

NORC invited all female panelists aged ≥18 years to provide informed consent and complete a brief eligibility screening survey. For panelists aged 15 to 17 years, NORC first obtained parental consent before inviting panelists to assent and participate. Participants received the equivalent of US $8 in NORC’s AmeriSpeak points. NORC provided deidentified data to the research team. The study protocol was approved by the Committee for the Protection of Human Subjects at the University of California, Berkeley (2021-02-14025) and the institutional review board of NORC (21-09-468).

### Measures

#### Key Contraceptive Access Metrics

This paper focuses on 2 key population-level metrics of contraceptive access: a conventional metric reflecting use of effective contraception, which is prioritized in the HP2030 contraception-related objectives, and a person-centered metric, reflecting use of preferred method of contraception [53]. For this analysis, we created both metrics to be as closely comparable as possible on key data inputs (age range and gender) and sample exclusions (fecundity and pregnancy status) while highlighting where they intentionally differ in other data inputs (contraceptive use and preferences and sexual activity; Multimedia Appendix 2). While HP2030 includes 2 metrics that separately focus on those aged 15 to 19 years and 20 to 44 years, our comparative analysis included the full age range included in the data set (ages of 15-44 years) to align with the working group’s priority of advancing metrics that were applicable across the spectrum of age and experiences.

The conventional metric, approximating the HP2030 approach to the extent possible with our data, reflects the use of effective contraception among survey respondents presumed at risk of unintended pregnancy. The numerator is the number of individuals using a method classified by the Centers for Disease Control and Prevention [16] as most or moderately effective for pregnancy prevention in the last month (vasectomy, implant, IUD, injectable contraception, oral contraceptive pills, the patch, the ring, or the diaphragm). The denominator includes individuals assigned female sex at birth who report ever having had penile-vaginal sex and are not currently pregnant or seeking pregnancy. This construction differs slightly from the HP2030 approach due to data availability. Our survey did not assess postpartum status, an exclusion criterion for the HP2030 metrics [5], and individuals assigned female sex at birth personally using permanent contraception were not eligible for this survey, although they are included in the HP2030 metrics. In addition, while the HP2030 denominators focuses on “women,” we use assignment of female sex at birth, as the HP2030 measurement details do not describe how gender is assessed [17].

The person-centered metric, use of preferred contraceptive method, reflects a desire to maintain current contraceptive use among current and prospective contraceptive users. The numerator is the number of current contraceptive users who do not want to switch to another or no method or stop using their method as soon as possible. We asked respondents whether they would rather use a (different) method of birth control (*yes*, *no*, or *unsure*); notably, some respondents who said that they would rather use a different method indicated that they would prefer to not be using a method altogether [53]. We developed and refined this question based on interviews with stakeholders, a literature review, an expert review, and cognitive interviews in English and Spanish. In addition, we also asked current contraceptive method users whether they would like to stop using each of their reported methods in the following year (*yes*, *no*, or *maybe*); those who indicated that they would like to stop using a method as soon as possible were not classified as using their preferred method, including multiple-method users who indicated a desire to discontinue use of any of their methods immediately. The denominator includes individuals who are current or prospective contraceptive users who are not pregnant or trying to become pregnant; the working group identified this population as having a self-identified need for contraception and related services as it includes (1) current users whose needs may include maintaining use of their current method, switching to a different method, or discontinuing use of their current method; and (2) prospective contraceptive users, that is, individuals not using contraception who indicated that there was a method they would like to use. Excluded are content nonusers—individuals not using a method simply because they do not want to; while this reflects a successful enactment of contraceptive preferences, these content nonusers are not considered to have a self-identified need for contraception. Notably, this metric allows for contraceptive use for reasons beyond pregnancy prevention. Details on the construction of this metric are described in greater depth elsewhere [53].

#### Key Demographic and SRH Characteristics

We focused on key sociodemographic and SRH characteristics to understand who is included and excluded in the 2 metrics’ denominators. Sociodemographic characteristics include age, racial and ethnic identity, health insurance status, sexual orientation, and gender identity. SRH characteristics include experience with penile-vaginal sex (*never had*, *had in the last year*, or *had more than a year ago*). We asked respondents who indicated that they may or would like to become pregnant in the future what the ideal timing would be (*in the next year*, *more than a year from now*, or *don’t know*). We examined current contraceptive method use based on whether respondents reported using any methods in the previous month. We describe all contraceptive methods that respondents reported using in the previous month, including use of multiple methods. We also created a mutually exclusive variable focused on the most effective method that respondents were using and report preference to stop use of this method in the following year overall, including as soon as possible. We describe whether current contraceptive users (1) were using their preferred method, (2) wanted to use a different or no method or stop using any of their methods as soon as possible, and (3) were uncertain about using a different method. We describe whether individuals not currently using contraception were (1) content nonusers (who do not report wanting to use a method), (2) prospective users (nonusers who indicate that there is a method that they want to use), and (3) uncertain nonusers (unsure whether they want to use a method).

### Analytic Approach

#### Overview

NORC constructed survey weights to account for differences between the sample and the US population. All analyses used the *svy* commands in Stata (version 17.0; StataCorp) to account for weighted data and complex survey design [57]. All reported proportions are weighted.

#### Sociodemographic and SRH Characteristics of the Sample Overall and by Metric

Our analytic sample (unweighted N=2760) excluded respondents who were pregnant or trying to become pregnant as they were not included in either metric, as well as respondents who were missing data on current contraceptive use. First, we present descriptive statistics for sociodemographic and SRH characteristics for the survey sample overall and for the subsamples meeting inclusion criteria for the 2 contraceptive access metrics examined in this analysis (heretofore referred to as the “conventional metric denominator” for the effective method use metric and the “person-centered metric denominator” for the preferred method use metric). Estimates slightly differ from those in previously published work due to different sample constructions when accounting for missing data in multivariable analyses [53].

#### Contraceptive Use and Preferences Among Individuals Included in the Conventional Metric Denominator

We examined contraceptive preferences among individuals meeting the inclusion criteria for the conventional metric denominator (those considered by HP2030 as being at risk of unintended pregnancy). Because this metric, as used in HP2030 and other public health contraceptive initiatives, implicitly frames use of most or moderately effective methods as successful, we leveraged our data set to examine whether those successful individuals’ preferences actually aligned with that assumption vis-à-vis desire to maintain current contraceptive use or nonuse and discontinue use of the current most effective method. Similarly, we examined individuals’ contraceptive preferences among those using other or no methods (the implied unsuccessful groups in the HP2030 approach).

#### Convergence and Divergence of the Denominators of the Conventional and Person-Centered Metrics

**Figure 1:**
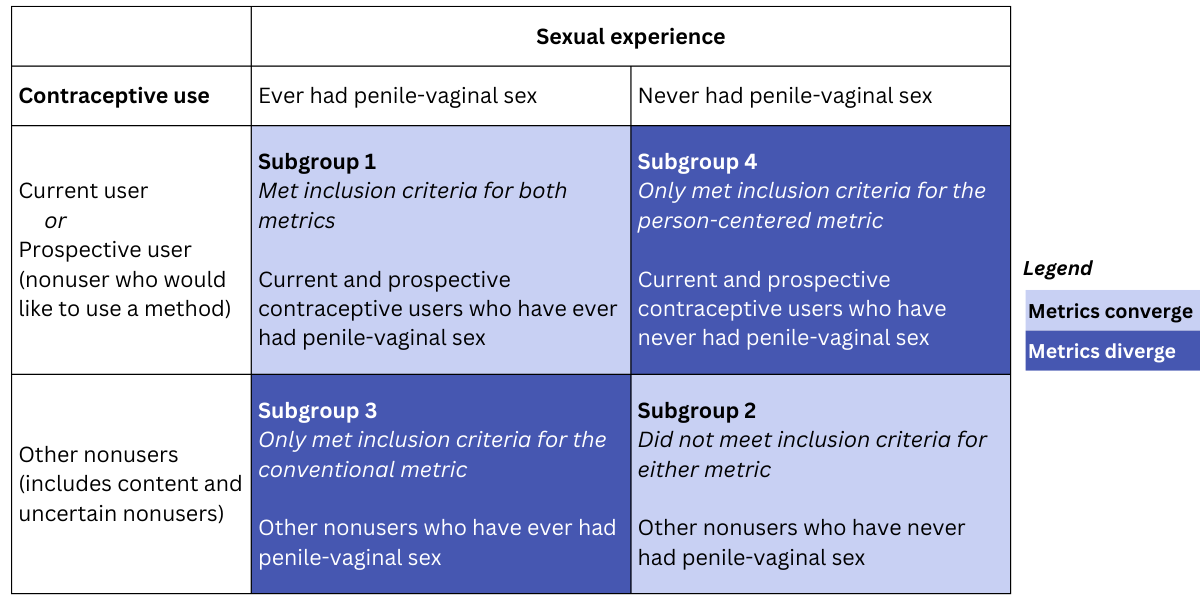
Convergence and divergence of 2 contraceptive metrics across contraceptive use and sexual experience of individuals aged 15 to 44 years assigned female sex at birth (2022).

We conducted a comparative analysis to highlight the differences between the conventional measurement approach (ie, use of effective method) and a person-centered measurement approach (ie, use of preferred method). For this analysis, we first determined whether each survey respondent met the inclusion criteria for the conventional and person-centered metrics (ie, would be included in each metric’s denominator). We classified respondents as being in 1 of 4 subgroups according to the 2 features that distinguish these metrics’ denominators (Figure 1): ever having had penile-vaginal sex (conventional metric inclusion criterion), and whether one is a current or prospective contraceptive user (person-centered metric inclusion criterion). The metrics converged when individuals were included in (subgroup 1) or excluded from (subgroup 2) both denominators. The metrics diverged if individuals were only included in the conventional metric denominator (subgroup 3) or the person-centered metric denominator (subgroup 4). As our comparative analysis sought to elucidate where the 2 metrics converged and diverged, we describe the distribution of age and SRH characteristics across the 4 subgroups. For the 2 divergent subgroups (3 and 4), we examined differences in age and ideal pregnancy timing using Rao-Scott–corrected chi-square tests. We also examined the distribution of race and ethnicity, insurance status, and gender; as there were no statistically significant differences, we do not present these results. Chi-square tests were not used for the other variables (sexual activity, current contraceptive use, and desire to use another contraceptive method) because each of these cross-tabulations had a structural 0 cell size; that is, there were no respondents reflected in the cell due to the inclusion requirements for the subgroups.

## Results

### Sociodemographic and SRH Characteristics Overall and by Metric

Sociodemographic and SRH characteristics are presented in Tables 1 and 2. The conventional metric denominator was slightly larger than the person-centered metric denominator (79.3% vs 74.3% of the overall sample, respectively; Table 1). The denominators were similar in distribution across demographic characteristics. Given the focus on pregnancy prevention, no respondents included in the conventional metric denominator reported never having penile-vaginal sex compared to 10% of those included in the person-centered metric denominator (Table 2). Contraceptive method mix and desire to use another method varied by metric. Most included in the person-centered metric were using contraception (92% compared to 79.3% of those included in the conventional metric denominator). More respondents included in the person-centered metric were current contraceptive users who wanted to use a different or no method or discontinue using a method immediately (20.8% compared to 18.2% included in the conventional metric). With regard to not using contraception, 12.8% of those included in the conventional metric were content nonusers compared to 0% of those included in the person-centered metric. Nonusers who wanted to use contraception (ie, prospective users) comprised a smaller proportion of the conventional metric denominator (4.3%) compared to the person-centered metric denominator (8%).

**Table 1:** Sociodemographic characteristics of individuals aged 15 to 44 years assigned female at birth, overall and for the conventional and person-centered contraceptive access metric denominators (2022; unweighted N=2760)^a^.

Sociodemographic characteristics		Full analytic sample^b^, unweighted n (weighted %)	Conventional metric denominator (unweighted n=2393)^c^, unweighted n (weighted %)	Person-centered metric denominator (unweighted n=2132)^d^, unweighted n (weighted %)
**Age group (y)**				
	15-17	201 (12.78)	64 (5.37)	91 (8)
	18-24	296 (27.51)	226 (25.15)	226 (28.15)
	25-29	574 (20.22)	503 (22.37)	470 (21.71)
	30-34	637 (17.24)	597 (20.54)	518 (19.04)
	35-39	598 (12.41)	563 (14.52)	476 (13.08)
	40-44	454 (9.83)	440 (12.05)	351 (10.01)
**Race and** **ethnicity**				
	Asian or Pacific Islander only	185 (7.15)	141 (6.24)	140 (6.26)
	Black only	386 (14.62)	317 (14.46)	258 (12.98)
	Latinx or Hispanic only	489 (21.4)	417 (20.84)	391 (22.14)
	Multiracial, not including Latinx or Hispanic	137 (3.3)	122 (3.59)	104 (3.35)
	White only	1526 (52.82)	1364 (54.18)	1214 (54.68)
	Another race or ethnicity only	37 (0.72)	32 (0.69)	25 (0.59)
**Insurance type**				
	Commercial (eg, employer based, direct purchase, or health insurance exchange)	1884 (63.24)	1663 (63.44)	1491 (64.59)
	State Medicaid or CHIP^e^	460 (19.44)	402 (21.26)	358 (20.39)
	Other public insurance (including Medicare, military or VA^f^, or IHS^g^)	115 (4.42)	100 (4.35)	84 (4.46)
	None	174 (6.45)	154 (6.6)	129 (6.06)
	Do not know	102 (5.23)	56 (3.36)	56 (3.73)
	Missing	25 (1.22)	18 (0.99)	14 (0.77)
**Current sexual orientation**				
	Straight or heterosexual	2287 (78.11)	2040 (81.27)	1816 (80.47)
	Gay or lesbian	61 (3)	35 (1.75)	18 (1.21)
	Bisexual	285 (13.5)	241 (13.86)	224 (14.55)
	Queer	58 (2.16)	45 (1.75)	42 (1.93)
	Something else	54 (2.57)	22 (0.94)	21 (1.37)
	Missing	15 (0.66)	10 (0.42)	11 (0.47)
**Current gender identity**				
	Woman	2682 (96.64)	2338 (97.22)	2078 (96.8)
	Man	10 (0.37)	7 (0.26)	7 (0.34)
	Transgender	2 (0.25)	1 (0.1)	0 (0)
	Genderqueer or nonbinary	38 (1.36)	26 (0.93)	23 (1.15)
	>1 gender	17 (1.01)	12 (1.07)	14 (1.24)
	Missing	11 (0.38)	9 (0.42)	10 (0.47)

^a^The analytic sample was limited to those who were not sterile, not pregnant or trying to become pregnant, and not missing data on current contraceptive use. The conventional metric was use of most or moderately effective contraceptive methods; the denominator was restricted to those who had ever had penile-vaginal sex. The person-centered metric was current use of preferred contraceptive method; the denominator was restricted to current contraceptive users and prospective users (individuals not using contraception but who would like to use it). Individuals may be included in both the conventional and person-centered metric denominators; therefore, the denominators for these metrics are not mutually exclusive.

^b^Population size estimate: 43,942,191.

^c^79.32% of full sample; population size estimate: 34,853,000.

^d^74.29% of full sample; population size estimate: 32,642,737.

^e^CHIP: Children’s Health Insurance Program.

^f^VA: Veterans Affairs.

^g^IHS: Indian Health Service.

**Table 2:** Sexual and reproductive health characteristics of individuals aged 15 to 44 years assigned female at birth overall and for the conventional and person-centered contraceptive access metric denominators (2022)^a^.

Sexual and reproductive health characteristics					Full analytic sample, unweighted n (weighted %)			Conventional metric denominator, unweighted n (weighted %)			Person-centered metric denominator, unweighted n (weighted %)
**Sexual activity**											
	Never had penile-vaginal sex			353 (20)			0 (0)			144 (10.03)	
	Had penile-vaginal sex in the last year			2097 (69.14)			2097 (87.17)			1839 (82.26)	
	Had penile-vaginal sex more than a year ago			280 (9.77)			280 (12.32)			128 (6.6)	
	Had penile-vaginal sex; missing timing of last sexual encounter			16 (0.4)			16 (0.51)			10 (0.34)	
	Missing			14 (0.69)			0 (0)			11 (0.76)	
**Ideal time to become pregnant**											
	In the next year			304 (7.93)			293 (9.55)			232 (8.27)	
	More than a year from now			783 (36.93)			659 (36.5)			591 (36.86)	
	Does not know			612 (22.8)			513 (21.66)			428 (20.76)	
	Does not ever want to become pregnant			1054 (32.07)			925 (32.07)			875 (33.79)	
	Missing			7 (0.28)			3 (0.21)			6 (0.32)	
**Currently using a contraceptive method^b^**											
	**Yes**			1988 (68.33)			1878 (79.29)			1988 (91.99)	
		Using preferred contraceptive method^c,d^	1294 (43.9)			1225 (51.01)			1294 (59.09)		
		Wants to use a different or no method or stop using any current method as soon as possible	423 (15.47)			403 (18.24)			423 (20.83)		
		Uncertain user (unsure whether they prefer using a different or no method)	266 (8.81)			246 (9.89)			266 (11.8)		
	**No**			772 (31.67)			515 (20.71)			144 (8.01)	
		Content nonuser (does not want to use contraception)	450 (17.65)			320 (12.8)			0 (0)		
		Prospective user (nonuser who wants to use contraception)	144 (5.95)			99 (4.26)			144 (8.01)		
		Uncertain nonuser (unsure whether they want to use contraception)	174 (7.79)			95 (3.64)			0 (0)		
Currently using multiple contraceptive methods					601 (24.03)			582 (29.56)			601 (32.34)
**Current contraceptive method use^e^**											
	Withdrawal or pulling out			639 (25.58)			621 (31.58)			639 (34.44)	
	Oral contraceptive pill			549 (21.92)			487 (23.4)			549 (29.51)	
	External condoms			478 (18.14)			468 (22.27)			478 (24.42)	
	Hormonal IUD^f^			331 (10.57)			322 (12.56)			331 (14.22)	
	Fertility awareness			207 (6.72)			199 (7.96)			207 (9.04)	
	Implant			108 (4.51)			101 (5.34)			108 (6.07)	
	Vasectomy			220 (4.42)			216 (5.48)			220 (5.94)	
	Copper IUD			77 (2.18)			75 (2.68)			77 (2.94)	
	Shot			52 (2.03)			50 (2.46)			52 (2.73)	
	Ring			39 (1.35)			37 (1.65)			39 (1.81)	
	Emergency contraception			35 (1.36)			33 (1.64)			35 (1.83)	
	Internal condoms			12 (0.69)			10 (0.78)			12 (0.93)	
	Patch			14 (0.55)			12 (0.61)			14 (0.74)	
	Spermicide			10 (0.22)			9 (0.27)			10 (0.3)	
Using any most or moderately effective method^c^					1324 (45.38)			1237 (51.64)			1324 (61.09)

^a^The analytic sample was limited to those who were not sterile, not pregnant or trying to become pregnant, and not missing data on current contraceptive use. The conventional metric was use of most or moderately effective contraceptive methods; the denominator was restricted to those who had ever had penile-vaginal sex. The person-centered metric was current use of preferred contraceptive method; the denominator was restricted to current contraceptive users and prospective users (individuals not using contraception but who would like to use it). Individuals may be included in both the conventional and person-centered metric denominators; therefore, the denominators for these metrics are not mutually exclusive.

^b^9 respondents (unweighted n) were missing the contraceptive use or nonuse subtype.

^c^One of the 2 key contraceptive access metrics.

^d^For multiple method users, this metric reflects whether the individual wants to maintain use of all their methods.

^e^Participants could report the use of multiple methods in the previous month.

^f^IUD: intrauterine device.

Table 2 includes the key contraception access metrics, with 51% of respondents included in the conventional metric classified as using their preferred method (compared to 59.1% of respondents included in the person-centered metric). Just over half (51.6%) of respondents included in the conventional metric denominator were using a most or moderately effective contraceptive method, compared to 61.1% of respondents included in the person-centered metric.

### Contraceptive Use and Preferences Among Those Included in the Conventional Metric Denominator

In Table 3, we present data on contraceptive use and preferences among those included in the conventional metric denominator of individuals who had ever had penile-vaginal sex who were not pregnant or seeking pregnancy. Approximately half (51.6%) were using a contraceptive method rated as most or moderately effective for pregnancy prevention. More than a quarter (27.7%) were using other contraceptive methods, whereas 20.7% were not using contraception. We examined the distribution of preferred method use, content nonuse, prospective use, and desired discontinuation in the following year by these 3 categories (most or moderately effective method use, other method use, and no contraceptive use). Among those using most or moderately effective contraceptive methods, 69.2% were using their preferred method, compared to 55.6% of those using another contraceptive method. Among those not using contraception, most (61.8%) were content nonusers, whereas 20.6% were prospective users who were interested in using contraception. Regarding discontinuation, among those using contraception, a majority of both users of most or moderately effective methods and users of other methods did not report a desire to stop use of their most effective method in the following year. However, there were still sizable proportions of individuals in both groups who indicated a clear or possible desire to stop using their current most effective method in the following year—11.8% of most or moderately effective method users and 20.4% of users of other methods had clear desires to discontinue, whereas 19.6% of most or moderately effective method users and 27.1% of other method users indicated that they might want to discontinue.

**Table 3:** Current contraceptive use and desires to switch and discontinue method use among individuals aged 15 to 44 years assigned female at birth meeting the inclusion criteria for the conventional metric denominator (2022)^a^.

Contraceptive use and preferences			Using a most or moderately effective contraceptive method (unweighted n=1237)^b^, unweighted n (weighted %)		Using other contraceptive method (unweighted n=641)^c^, unweighted n (weighted %)		Not using contraception (unweighted n=515)^d^, unweighted n (weighted %)
Using preferred contraceptive method^e^			875 (69.2)		350 (55.6)		—^f^
Content nonuser (does not want to use contraception)			—		—		320 (61.82)
Prospective user (nonuser who wants to use contraception)			—		—		99 (20.59)
Uncertain nonuser (unsure whether they want to use contraception)			—		—		95 (17.59)
**Would like to stop using current most effective method in the next year^g^**							
	Yes	151 (11.82)		124 (20.35)		—	
	As soon as possible	37 (3.14)		41 (7.72)		—	
	No	853 (68.61)		338 (52.52)		—	
	Maybe	218 (19.57)		176 (27.12)		—	

^a^Respondents with missing data were excluded from cross-tabulations. The conventional metric was use of most or moderately effective contraceptive methods; the denominator was restricted to those who had ever had penile-vaginal sex.

^b^51.64% of those included in the conventional metric denominator; population size estimate: 17,997,224.

^c^27.65% of those included in the conventional metric denominator; population size estimate: 9,637,152.

^d^20.71% of those included in the conventional metric denominator; population size estimate: 7,218,624.

^e^For multiple method users, this metric reflects whether the individual wants to maintain use of all their methods.

^f^Category not applicable. Those not using contraception are not included in numerator of the preferred method use metric, which focuses on current users who want to maintain use of their methods. Those using a moderately or most effective method or another method are not nonusers.

^g^This represents a mutually exclusive variable describing whether respondents would like to stop using their most effective current contraceptive method. It is independent of the Centers for Disease Control and Prevention designation of most or moderately effective methods.

### Convergence and Divergence of the Denominators of the Conventional and Person-Centered Metrics

In our comparative analysis, we first examined where the denominators of the person-centered metric and conventional metrics converged and diverged by determining whether individuals would be included in the denominators for both metrics (subgroup 1), excluded from both (subgroup 2), included only in the conventional metric (subgroup 3), or included only in the person-centered metric (subgroup 4; Figure 1). We found alignment across the 2 metrics for most of the sample—66.3% of respondents were included in both metric denominators (subgroup 1), and 12.7% were excluded from both (subgroup 2; Table 4). These 2 subgroups represent an estimated nearly 35 million individuals. About 13% of the analytic sample met inclusion criteria only for the conventional metric denominator (population estimate: 5.7 million individuals; subgroup 3); this subgroup exclusively comprised content nonusers who had ever had penile-vaginal sex. Finally, subgroup 4 included 8% of the analytic sample included only in the person-centered metric denominator. Subgroup 4 included current or prospective contraceptive users who had never had penile-vaginal sex (population estimate: 3.5 million individuals); despite their current or desired contraceptive use, they did not meet inclusion criteria for the conventional metric denominator. Examining these 2 divergent subgroups highlights fundamental differences in assumptions of the metrics regarding who is seen as being in need of contraception.

**Table 4:** Convergence and divergence of age and sexual and reproductive health characteristics by inclusion in the conventional and person-centered contraceptive access metrics among individuals aged 15 to 44 years assigned female sex at birth (2022)^a^.

Age and sexual and reproductive health characteristics				Convergence between the 2 metrics^b^				Divergence between the 2 metrics^c^				
				Subgroup 1^d^: included in both metrics (unweighted n=1977), unweighted n (weighted %)		Subgroup 2^e^: excluded from both metrics (unweighted n=212), unweighted n (weighted %)		Subgroup 3^f^: included only in the conventional metric (unweighted n=416), unweighted n (weighted %)		Subgroup 4^g^: included only in the person-centered metric (unweighted n=155), unweighted n (weighted %)	*P* value (comparing subgroups 3 and 4)	
**Age group (y)**												<.001
	15-17		51 (5.34)		97 (48.27)		13 (5.55)		40 (30.03)			
	18-24		198 (26.82)		42 (34.99)		28 (16.64)		28 (39.09)			
	25-29		431 (22.55)		32 (10.24)		72 (21.41)		39 (14.79)			
	30-34		497 (20.54)		19 (3.3)		100 (20.53)		21 (6.62)			
	35-39		453 (13.67)		12 (1.8)		110 (18.87)		23 (8.23)			
	40-44		347 (11.08)		10 (1.4)		93 (16.98)		4 (1.23)			
**Sexual activity**												—^h^
	Never had penile-vaginal sex		0 (0)		209 (100)		0 (0)		144 (100)			
	Had penile-vaginal sex in the last year		1839 (92.21)		0 (0)		258 (61.56)		0 (0)			
	Had penile-vaginal sex more than a year ago		128 (7.40)		0 (0)		152 (37.31)		0 (0)			
	Had penile-vaginal sex; missing timing of last sexual encounter		10 (0.39)		0 (0)		6 (0.11)		0 (0)			
**Ideal time to become pregnant**												.02
	In the next year		225 (8.96)		4 (1.04)		68 (12.7)		7 (2.78)			
	More than a year from now		540 (36.78)		73 (38.83)		119 (35.57)		51 (38.68)			
	Does not know		387 (19.88)		58 (26.4)		126 (30.96)		41 (28.68)			
	Does not ever want to become pregnant		822 (34.38)		76 (33.73)		103 (20.78)		53 (29.87)			
**Currently using a contraceptive method^i^**												—
	**Yes**		1878 (94.9)		0 (0)		0 (0)		110 (67.93)			
		Using preferred contraceptive method^j^	1225 (61.05)		—		—		69 (42.91)			
		Wants to use different or no method or stop using any current method as soon as possible	403 (21.84)		—		—		20 (12.48)			
		Uncertain user (unsure whether they prefer using a different or no method)	246 (11.84)		—		—		20 (12.06)			
	**No**		99 (5.11)		212 (100)		416 (100)		45 (32.07)			
		Content nonuser (does not want to use contraception)	0 (0)		130 (59.25)		320 (77.78)		0 (0)			
		Prospective user (nonuser who wants to use contraception)	99 (5.10)		0 (0)		0 (0)		45 (32.07)			
		Uncertain nonuser (unsure whether they want to use contraception)	0 (0)		79 (38.73)		95 (22.13)		0 (0)			
Using any most or moderately effective method				1237 (61.81)		0 (0)		0 (0)		87 (55.19)	—	

^a^Respondents’ missing data were excluded from cross-tabulations.

^b^Convergence indicates that individuals were treated the same by both metrics, either included (subgroup 1) or excluded (subgroup 2) from both denominators.

^c^Divergence indicates that individuals were treated differently by the two metrics, only included in the conventional metric denominator (subgroup 3) or only included in the person-centered metric denominator (subgroup 4). Rao-Scott–corrected chi-square tests are presented to compare differences between the 2 divergent subgroups (3 and 4) for age and ideal time to become pregnant.

^d^66.27% of full analytic sample; population size estimate: 29,119,287.

^e^12.67% of full analytic sample; population size estimate: 5,565,741.

^f^13.05% of full analytic sample; population size estimate: 5,733,713.

^g^8.02% of full analytic sample; population size estimate: 3,523,450.

^h^Chi-square tests were not used for sexual activity, current contraceptive use, and current contraceptive use status because each of these cross-tabulations had a structural 0 cell size; that is, there were no respondents reflected in the cell due to the inclusion requirements for the subgroups.

^i^9 respondents (unweighted n) were missing the contraceptive use or nonuse subtype.

^j^For multiple method users, this metric reflects whether the individual wants to maintain use of all their methods.

There were differences in age and SRH experiences among the divergent subgroups (Table 4). Those included only in the person-centered metric denominator (subgroup 4) were disproportionately younger (eg, 69% were aged <25 years compared to 22.1% of those included only in the conventional metric denominator, subgroup 3). Regarding SRH experiences, the 2 divergent subgroups differed in history of penile-vaginal sex (a requirement for inclusion in the conventional metric). All respondents (100%) included only in the conventional metric denominator had previously had penile-vaginal sex, whereas no one included only in the person-centered metric denominator reported ever having penile-vaginal sex. More respondents included only in the conventional metric denominator expressed a desire to become pregnant in the following year (12.7%) compared to those included only in the person-centered metric denominator (2.8%).

Although excluded from the conventional metric, most individuals (67.9%) included only in the person-centered metric reported current contraceptive use. No individuals included only in the conventional metric denominator were prospective users compared to 32.1% of those included only in the person-centered metric denominator. Most respondents included only in the conventional metric were content nonusers (77.8%). For subgroup 4 (included only in the person-centered metric denominator), 42.9% of the respondents were current contraceptive users using their preferred method, whereas more than half (55.2%) were using a most or moderately effective method (primarily contraceptive pills or hormonal IUDs; data not shown).

## Discussion

### Principal Findings

In our comparative analysis, we highlighted differences in who meets the inclusion criteria for the conventional metric focused on effective contraceptive method use and for a person-centered metric focused on use of preferred contraceptive method, demonstrating numerous limitations with and assumptions of the conventional approach. Most importantly, we found that 13% of individuals included in the conventional metric denominator expressly did not want to use contraception (translating to an estimated nearly 4.5 million individuals). Furthermore, given the narrow focus on pregnancy prevention as the key driver for understanding contraceptive access, the conventional metric excludes many who are currently using or want to use contraception but are not considered at risk of unintended pregnancy because they have never had penile-vaginal sex. At the population level, as highlighted by our analysis of divergence between the 2 metrics, this translates into an estimated >9 million individuals who may not be accurately represented by one of the most common contraceptive access metrics in the United States.

This analysis highlights the implications of the assumptions of the HP2030 inclusion criteria. First, the denominator for the conventional metric includes individuals whose behaviors are aligned with their desires—they are not using contraception, nor do they want to use contraception. These content or autonomous nonusers’ [29] preferences are explicitly ignored when those who develop or set contraceptive programs or policies deem this group to be unsuccessful in the metric of effective contraceptive use, essentially targeting this group’s contraceptive behavior as needing to be changed from nonuse to use. Although person-centered data on autonomous nonuse are lacking, we know that some people may not have a found a method that meets their needs [19] or may be open to the possibility of pregnancy [20], and others may feel that abortion is an acceptable and feasible outcome should they unexpectedly become pregnant [58]. Second, the denominator for the conventional metric excludes individuals who have never had penile-vaginal sex, implying that they have no need for contraception because they are presumed to not be at risk of unintended pregnancy and overlooking broad evidence indicating that people use contraception for a variety of reasons, including but not limited to pregnancy prevention. For example, in 2022, a total of 39% of adult female contraceptive users in the United States used their method for a reason beyond just pregnancy prevention, such as menstruation management, managing a medical condition, or prevention of sexually transmitted infections [20]. Our findings bolster this evidence base; higher levels of contraceptive use among individuals included in the person-centered metric compared to those included in the conventional metric highlight that the former is more broadly inclusive of the range of individuals using contraception for any reason and that the latter is missing people who are using or want to use contraception. Centering SRH equity in contraceptive access and public health goals ensures that everyone who self-identifies a possible need for contraception can obtain it and any related services (including contraceptive care to fulfill the need to switch and discontinue use of methods).

Individuals’ preferences for switching and discontinuing their methods further reveal the limitations of the conventional approach used in HP2030. We found that, among users of a most or moderately effective method within the conventional metric sample, over a quarter were not classified as using their preferred method, and almost one-third wanted to potentially or definitely stop using their current method within a year. This finding highlights another limitation of the conventional metric approach, which implicitly frames use of effective contraceptive methods as successful—focusing on use of these specific methods without accounting for preferences or recognizing the dynamic nature of contraceptive use masks the contraceptive needs of this purportedly successful group. Moreover, among those using other contraceptive methods, more than half were using their preferred method; among those using no method, 62% did not want to use contraception. Importantly, although there were lower rates of desired switching and discontinuation among those using most or moderately effective methods compared to those using other methods or no methods, these percentages still translated to larger overall population estimates with unfulfilled preferences within the group of individuals using most or moderately effective methods. This highlights another key limitation of the conventional focus on increasing use of effective contraception: those considered unsuccessful because they are not using a most or moderately effective contraceptive method are often enacting their preferences. Therefore, targeting them for increased use undermines reproductive autonomy and does not align with SRH equity.

Our comparative analysis highlights the importance of integrating contraceptive preferences into metrics to monitor contraceptive need and access and inform policy and program strategies, both for ensuring appropriate access to high-quality services and to advance SRH equity. Strengths of this work include the intentional and diverse input and feedback that contributed to shaping the survey design and, especially, the preferred method use metric examined in this analysis. The person-centered focus of the survey allowed us to examine contraceptive preferences within the sample, highlighting a significant number of individuals who are misrepresented using the conventional metric approach. Inclusion of items regarding preferences for contraceptive initiation among nonusers and switching and discontinuation among current users revealed important insights about the assumptions of conventional approaches to public health goals and metrics that frame use of certain methods as a universal good. Finally, leveraging recent, nationally representative data to examine the 2 metrics in the comparative analysis allows us to broadly generalize our findings to today’s landscape of contraceptive access and how progress toward increased access is being measured at the national level.

### Comparison With Prior Work

While we are aware of no other studies focused on the United States that compare a conventional measurement approach to a person-centered one, prior work examining the concept of unmet need has similarly found substantial misclassification of individuals’ contraceptive needs when metrics are based on assumptions about who should be using contraception with no consideration of the preferences of these presumed users. Unmet need is a population-level metric, typically focused on women in the Global South, that ostensibly claims to identify the population that needs contraception [59]. This need is determined based on demographic characteristics (gender and age) and sexual behavior and neglects whether individuals want to use contraception. A study by Senderowicz and Maloney [60] used data from 7 sub-Saharan African countries and found that most individuals classified as having an unmet need for contraception did not express a desire to use contraception. In this same vein, a 1972 paper by Blake and Das Gupta [61] found that unmet need estimates misclassified 74% of the 4.6 million US women who were poor or near poor and presumed to have an unmet need for contraception.

In our analyses as well as in other research, people who do not identify as women or heterosexual report contraceptive use and preferences regarding use. Indeed, gender-expansive and queer individuals experience greater barriers to accessing contraception [30-33,62,63], so their contraceptive needs should be included, understood, and prioritized in any initiative seeking to integrate an SRH equity lens into ensuring contraceptive access. In addition, a broader focus beyond pregnancy prevention highlights the importance of including young people in contraceptive access metrics, including adolescents even aged <15 years, who are not represented in our data set or in the contraception-related Healthy People objectives but who may be using (or want to use) contraception for menstruation or acne management even if they are not sexually active [22].

### Limitations

Importantly, individuals assigned female sex at birth who personally use permanent contraception were not included in the survey sample, and thus, these findings cannot be generalized to this group of contraceptive users. Other research highlights that some permanent contraception users express a desire for their sterilization procedures to be reversed [64], which is important to give voice to even though permanent contraception is not a modifiable contraceptive method. The lower levels of use of most or moderately effectively methods in the conventional metric sample (52% among those aged 15-44 years) compared to national levels most recently cited in HP2030 (62% among those aged 20-44 years) likely reflect difference between the samples based on exclusion or inclusion of individuals who had undergone sterilization procedures [5]. As a result, our comparative analysis is not an exact estimation of the 2 different approaches with the full ideal populations for both metrics; still, this comparative analysis provides valuable information about the assumptions of the conventional approach. The person-centered metric is not without limitations [53]. It represents contraceptive use preferences at one moment in time, whereas it may be useful for policy purposes to capture self-identified need over 12 months. In addition, the primary survey question to assess use of preferred method could be further refined by adding the timing of “right now” to the primary question to ensure that individuals desire to use these methods currently and using follow-up questions to understand uncertain responses.

### Conclusions

Uptake of methods highly effective for pregnancy prevention, such as long-acting reversible contraception, is frequently cast as a success for contraceptive programs and clinical practice [42]. However, this framing—reflected in the HP2030 objectives and associated metrics that emphasize use of most or moderately effective methods for pregnancy prevention—neglects individuals’ contraceptive preferences, resulting in programs and policies that do not reflect the priorities of the individuals they seek to serve. Importantly, the population estimates for the 2 metrics were relatively similar, suggesting that the person-centered approach does not significantly decrease the estimated population potentially in need of contraception but rather more precisely identifies it.

Building programs and policies around public health goals and metrics that are not aligned with priorities and preferences of those reflected in the measures is, at best, ineffective and wasteful and, at worst, in violation of people’s autonomy and misaligned with SRH equity. We do not have to look too far back in history to identify examples of how programs or policies that ostensibly were set up in service of increasing contraceptive access veered from this objective and toward problematic justifications for increasing use of specific, effective methods for certain low-income populations via poverty reduction arguments [39,65,66]. These examples demonstrate how even the seemingly benign and objective act of constructing metrics is not without subjectivity and can perpetuate inequities rather than help reduce or eradicate them.

National public health objectives and metrics focused on contraceptive access should be informed by SRH equity and center people’s preferences regarding which methods they choose to use and be value neutral about these choices [1]; current metrics that set goals around use of effective methods meet neither of these criteria and, instead, embed externally set assumptions about which methods are best. Healthy People reflects the public health goals of the United States and is just one of many initiatives that could benefit from a close examination of its objectives and related metrics for alignment with the principles of SRH equity. Our results suggest possibilities for Healthy People and contraceptive access efforts broadly to align program and policy efforts with SRH equity to support people in achieving reproductive autonomy and guard against efforts that perpetuate reproductive injustices.

## Appendix

Multimedia Appendix 1 Checklist for Reporting Results of Internet E-Surveys.

Multimedia Appendix 2 Data inputs and exclusions for 3 metrics to examine contraceptive access drawing on an existing population-level metric used in Healthy People 2030 and 2 metrics used in this analysis.

## Abbreviations

HP2030: Healthy People 2030
IUD: intrauterine device
SRH: sexual and reproductive health

## Data Availability

The data sets analyzed during this study will be available in the Open Science Framework repository [67].
