# Supplementary material for: Integrating Sexual and Reproductive Health Equity Into Public Health Goals and Metrics: Comparative Analysis of Healthy People 2030’s Approach and a Person-Centered Approach to Contraceptive Access Using Population-Based Data
Source: JMIR Public Health Surveill. 2024 Aug 20;10:e58009. doi: 10.2196/58009 (PMC11372330; doi:10.2196/58009)
Supplement: Multimedia Appendix 1 [file publichealth_v10i1e58009_app1.pdf]

Multimedia Appendix 1: Checklist for Reporting Results of Internet E-Surveys (CHERRIES)

Item Category	***Checklist Item***	***Explanation***	***Response***
Design	Describe survey design	Describe target population, sample frame. Is the sample a convenience sample? (In "open" surveys this is most likely.)	The target population, sample, and approach are described in the Methods section.
IRB approval and informed consent process	IRB approval	Mention whether the study has been approved by an IRB.	The Committee for the Protection of Human Subjects at the University of California, Berkeley and the institutional review board of NORC at the University of Chicago approved the study protocol.
	Informed consent	Describe the informed consent process. Where were the participants told the length of time of the survey, which data were stored and where and for how long, who the investigator was, and the purpose of the study?	We mention that informed consent, including parental consent and minor assent, were obtained. After screening, eligible participants were directed to an online informed consent form. The informed consent included the investigator, study purpose, survey length, and confidentiality/data storage. Respondents clicked "yes" to indicate consent, and then proceeded to the survey.
	Data protection	If any personal information was collected or stored, describe what mechanisms were used to protect unauthorized access.	All personally identifiable information (PII) submitted to NORC is kept on physically secure password-protected encrypted data storage systems within actively monitored network firewalls. NORC uses expandable Storage Area Networks (SANs), relationship database management systems, internet-enabled applications, high capacity back-up infrastructure, and virtual servers to manage data and maintain high security. NORC's security program is compliant with federal regulations, including NIST 800-53. All records at NORC containing personally-identifiable information (PII), such as names, addresses, and emails, are kept on physically secure password-protected, encrypted, partitioned data storage systems within tightly

Item Category	***Checklist Item***	***Explanation***	***Response***
			controlled firewalls. Access to data at NORC is restricted to the minimum necessary level and all software logins require an encrypted challenge/response technology.<br>At UC Berkeley, de-identified survey data was stored on password-protected computers of research staff. Any personal information (e.g., census tract) is stored on the Secure Research Data and Compute (SRDC) Platform, a secure computing environment for highly sensitive (P4) data.
Development and testing	Development and testing	State how the survey was developed, including whether the usability and technical functionality of the electronic questionnaire had been tested before fielding the questionnaire.	The survey was developed through a multi-year stakeholder engaged process introduced and cited. The survey was tested by research staff and NORC staff multiple times before fielding.
Recruitment process and description of the sample having access to the questionnaire	Open survey versus closed survey	An "open survey" is a survey open for each visitor of a site, while a closed survey is only open to a sample which the investigator knows (password-protected survey).	The survey was fielded by NORC, using their AmeriSpeak panel. Only AmeriSpeak panel members who met age and sex criteria were invited to participate.
	Contact mode	Indicate whether or not the initial contact with the potential participants was made on the Internet. (Investigators may also send out questionnaires by mail and allow for Web-based data entry.)	NORC invited potential participants via email, which included a link to the screening survey. The same information was available in invited panelists' web portal and mobile apps.
	Advertising the survey	How/where was the survey announced or advertised? Some examples are offline media (newspapers), or online (mailing lists – If yes, which ones?) or banner ads (Where were these banner ads posted and what did they look like?). It is important to know the wording of the announcement as it will heavily influence who chooses to participate. Ideally the	NORC invited potential participants from their AmeriSpeak panel using their standard communication approach.

Item Category	*Checklist Item*	*Explanation*	*Response*
		survey announcement should be published as an appendix.	
Survey admin-istration	Web/E-mail	State the type of e-survey (eg, one posted on a Web site, or one sent out through e-mail). If it is an e-mail survey, were the responses entered manually into a database, or was there an automatic method for capturing responses?	NORC invited panelists via email). Interested panelists could take the survey online by following the link in the invitation email or accessing the link in their AmeriSpeak mobile app or web portal.
	Context	Describe the Web site (for mailing list/newsgroup) in which the survey was posted. What is the Web site about, who is visiting it, what are visitors normally looking for? Discuss to what degree the content of the Web site could pre-select the sample or influence the results. For example, a survey about vaccination on a anti-immunization Web site will have different results from a Web survey conducted on a government Web site	Respondents are members of the AmeriSpeak panel, and therefore are accustomed to receiving survey invitations from NORC in this manner.
	Mandatory/voluntary	Was it a mandatory survey to be filled in by every visitor who wanted to enter the Web site, or was it a voluntary survey?	The survey was voluntary.
	Incentives	Were any incentives offered (eg, monetary, prizes, or non-monetary incentives such as an offer to provide the survey results)?	Respondents received 8,000 points (the standard AmeriSpeak incentive), which is the equivalent of approximately $8.
	Time/Date	In what timeframe were the data collected?	January-March 2022
	Randomization of items or questionnaires	To prevent biases items can be randomized or alternated.	For select questions, response items were randomized in groups. None of these questions are included in the analyses for this manuscript.
	Adaptive questioning	Use adaptive questioning (certain items, or only conditionally displayed based on responses to other items) to reduce number and complexity of the questions.	We used adaptive questioning in the survey to ensure respondents only received questions that were relevant to them. For example, only those who indicated they were currently using the oral

Item Category	*Checklist Item*	*Explanation*	*Response*
			contraceptive pill were asked any questions about their experiences using the pill.
	Number of Items	What was the number of questionnaire items per page? The number of items is an important factor for the completion rate.	NORC programmed and managed the online survey, so we do not have the exact number. However, due to our use of adaptive questioning, the number of items per page was not necessarily the same for each respondent.
	Number of screens (pages)	Over how many pages was the questionnaire distributed? The number of items is an important factor for the completion rate.	NORC programmed and managed the online survey, so we do not have the exact number. However, due to our use of adaptive questioning, the number of pages was not necessarily the same for each respondent.
	Completeness check	It is technically possible to do consistency or completeness checks before the questionnaire is submitted. Was this done, and if "yes", how (usually JAVAScript)? An alternative is to check for completeness after the questionnaire has been submitted (and highlight mandatory items). If this has been done, it should be reported. All items should provide a non-response option such as "not applicable" or "rather not say", and selection of one response option should be enforced.	After data collection, NORC applied quality controls to remove cases deemed to be poor quality. These included: speeding (less than 1/3 median duration); high refusal (skip/refuse >50% eligible questions); straight-lining grid series responses.
	Review step	State whether respondents were able to review and change their answers (eg, through a Back button or a Review step which displays a summary of the responses and asks the respondents if they are correct).	Respondents were able to return to the previous page. In addition, select questions included a summary review step.
Response rates	Unique site visitor	If you provide view rates or participation rates, you need to define how you determined a unique visitor. There are different techniques available, based on IP addresses or cookies or both.	Based on how NORC programs their surveys and invites panelists, AmeriSpeak panelists cannot take the survey more than once. Each panelist is identifiable to NORC based on a unique ID.

Item Category	*Checklist Item*	*Explanation*	*Response*
	View rate (Ratio of unique survey visitors/unique site visitors)	Requires counting unique visitors to the first page of the survey, divided by the number of unique site visitors (not page views!). It is not unusual to have view rates of less than 0.1 % if the survey is voluntary.	N/A
	Participation rate (Ratio of unique visitors who agreed to participate/unique first survey page visitors)	Count the unique number of people who filled in the first survey page (or agreed to participate, for example by checking a checkbox), divided by visitors who visit the first page of the survey (or the informed consents page, if present). This can also be called "recruitment" rate.	We do not have this rate from NORC.
	Completion rate (Ratio of users who finished the survey/users who agreed to participate)	The number of people submitting the last questionnaire page, divided by the number of people who agreed to participate (or submitted the first survey page). This is only relevant if there is a separate "informed consent" page or if the survey goes over several pages. This is a measure for attrition. Note that "completion" can involve leaving questionnaire items blank. This is not a measure for how completely questionnaires were filled in. (If you need a measure for this, use the word "completeness rate".)	NORC did not provide this rate. However, 97% of panelists who were screened and determined to be eligible went on to complete the survey.
Preventing multiple entries from the same individual	Cookies used	Indicate whether cookies were used to assign a unique user identifier to each client computer. If so, mention the page on which the cookie was set and read, and how long the cookie was valid. Were duplicate entries avoided by preventing users access to the survey twice; or were duplicate database entries having the same user ID eliminated before analysis? In the latter case, which entries were kept for analysis (eg, the first entry or the most recent)?	NORC assigns unique IDs to respondents; use of these IDs ensures there are no duplicates.
	IP check	Indicate whether the IP address of the client computer was used to identify potential duplicate entries from	NORC assigns unique IDs to respondents; use of these IDs ensures there are no duplicates.

Item Category	*Checklist Item*	*Explanation*	*Response*
		the same user. If so, mention the period of time for which no two entries from the same IP address were allowed (eg, 24 hours). Were duplicate entries avoided by preventing users with the same IP address access to the survey twice; or were duplicate database entries having the same IP address within a given period of time eliminated before analysis? If the latter, which entries were kept for analysis (eg, the first entry or the most recent)?	
	Log file analysis	Indicate whether other techniques to analyze the log file for identification of multiple entries were used. If so, please describe.	NORC has internal processes to assign unique IDs to respondents and ensure there are no duplicates.
	Registration	In "closed" (non-open) surveys, users need to login first and it is easier to prevent duplicate entries from the same user. Describe how this was done. For example, was the survey never displayed a second time once the user had filled it in, or was the username stored together with the survey results and later eliminated? If the latter, which entries were kept for analysis (eg, the first entry or the most recent)?	Access to surveys was controlled by NORC. Panelists have secure log-ins to NORC's web portal or app. The surveys are programmed to ensure panelists cannot take the survey more than once.
Analysis	Handling of incomplete questionnaires	Were only completed questionnaires analyzed? Were questionnaires which terminated early (where, for example, users did not go through all questionnaire pages) also analyzed?	Complete surveys included those where the respondent went through the entire survey and was not removed for quality concerns mentioned above. Respondents were permitted to skip questions or indicate they preferred not to answer. Those surveys were included in analyses unless otherwise indicated in the manuscript.
	Questionnaires submitted with an atypical timestamp	Some investigators may measure the time people needed to fill in a questionnaire and exclude questionnaires that were submitted too soon. Specify	NORC removed respondents deemed "speeders," which was defined as taking less than 1/3 of the median survey duration.

Item Category	Checklist Item	Explanation	Response
		the timeframe that was used as a cut-off point, and describe how this point was determined.	
	Statistical correction	Indicate whether any methods such as weighting of items or propensity scores have been used to adjust for the non-representative sample; if so, please describe the methods.	NORC constructed survey weights to account for differences between the sample and the U.S. population. Weights are based on age, education, race/ethnicity, marital status, number of children in household, and age by race/ethnicity.

This checklist has been modified from Eysenbach G. Improving the quality of Web surveys: the Checklist for Reporting Results of Internet E-Surveys (CHERRIES). J Med Internet Res. 2004 Sep 29;6(3):e34 [erratum in J Med Internet Res. 2012; 14(1): e8.]. Article available at https://www.jmir.org/2004/3/e34/; erratum available https://www.jmir.org/2012/1/e8/. Copyright ©Gunther Eysenbach. Originally published in the Journal of Medical Internet Research, 29.9.2004 and 04.01.2012. This is an open-access article distributed under the terms of the Creative Commons Attribution License (https://creativecommons.org/licenses/by/2.0/), which permits unrestricted use, distribution, and reproduction in any medium, provided the original work, first published in the Journal of Medical Internet Research, is properly cited.
