# Supplementary material for: Integrating Sexual and Reproductive Health Equity Into Public Health Goals and Metrics: Comparative Analysis of Healthy People 2030’s Approach and a Person-Centered Approach to Contraceptive Access Using Population-Based Data
Source: JMIR Public Health Surveill. 2024 Aug 20;10:e58009. doi: 10.2196/58009 (PMC11372330; doi:10.2196/58009)
Supplement: Multimedia Appendix 2 [file publichealth_v10i1e58009_app2.pdf]

	**Existing population-level metric**	**Population-level metrics used in this analysis**	
	Use of effective contraception, as it appears in Healthy People 2030	*Use of effective contraception, approximating the conventional metric approach used in Healthy People 2030*	*Use of preferred contraceptive method, a person-centered metric of contraceptive access*
Data inputs			
Age range	20-44	15-44	15-44
Gender	"Women"	Assigned female sex at birth	Assigned female sex at birth
Current contraceptive use	Use of permanent contraception (tubal, vasectomy), IUDs, implants, shot, pills, patch, ring, diaphragm in the last month	Use of vasectomy, IUDs, implants, shot, pills, patch, ring, diaphragm in the last month	N/A
Current use of preferred contraceptive method(s)	N/A	N/A	Desire to maintain current contraceptive method use (desire to switch methods or stop method use ASAP is excluded); all methods included
Sexual activity	Ever had sex with a "man"	Ever had penile-vaginal sex	N/A
Sample exclusions			
Fecundity	Not sterile for surgical non-contraceptive or non-surgical reasons	Using sterilization (tubal) or known to be sterile	Using sterilization (tubal) or known to be sterile
Pregnancy status	Not pregnant<br>Not seeking pregnancy<br>Not postpartum	Not pregnant<br>Not seeking pregnancy	Not pregnant<br>Not seeking pregnancy
Metric construction			
Numerator	Women ages 20-44 using most/moderately effective method in the last month	Individuals assigned female sex at birth ages 15-44 using most/moderately effective method in the last month	Individuals assigned female sex at birth ages 15-44 using desired contraceptive method in the last month
Denominator	Women ages 20-44 at risk of unintended pregnancy*	Individuals assigned female sex at birth ages 15-44 at risk of unintended pregnancy	Individuals assigned female sex at birth ages 15-44 who are current or prospective contraceptive users

Notes : Another Healthy People 2030 objective focuses on 15-19-year-olds (with the same data inputs). The Healthy People 2030 measurement details do not describe how gender is measured. * Risk for unintended pregnancy in Healthy People 2030 is based on ever having sex with a "man" and the sample exclusions.
